# Implications of oral biofilms in medically at risk persons^☆^

**DOI:** 10.1016/S1674-8301(12)60001-3

**Published:** 2012-01

**Authors:** Kevin H.-K. Yip, Roger J. Smales

**Affiliations:** a School of Dentistry, Charles Sturt University, Orange, New South Wales, Australia;; b College of Stomatology, Nanjing Medical University, Nanjing, Jiangsu 210029 China;; c School of Dentistry, Faculty of Health Sciences, the University of Adelaide, Adelaide, Australia.

**Keywords:** oral biofilm, aspiration pneumonia, coronary heart disease

## Abstract

There is the need to understand the composition of oral biofilms so that appropriate preventive and treatment regimens, including using appropriate antimicrobials, can be developed further. Additionally, when the systemic effects from specific microorganisms in oral biofilms are better understood, more targeted preventive treatment options may be recommended for persons at high risk for potential systemic diseases such as cardiovascular disease, and for aspiration pneumonia. Hence, the possible association between periodontopathic microorganisms, and also between cariogenic microorganisms in high caries risk persons, and systemic diseases requires further research involving metagenomic and large well-designed clinical studies. Effective preventive oral care is important for reducing potential systemic diseases.

## INTRODUCTION

The significant relationship between oral and general health is increasingly recognized by both medical and dental health care personnel[1]. Persons with compromised general health from chronic diseases, and perhaps also their associated treatments, are at high risk for oral disease. In addition, as has been reviewed elsewhere[1]-[3], several epidemiological and many clinical reports have linked, to varying degrees, oral infections and their products of inflammation with increasing rates of septicemia and several cardiovascular, central nervous system, respiratory and skeletal infections. Poor glycemic control in diabetics, and low-weight preterm births perhaps may also be associated with poor plaque control and resultant poor oral health. Unlike other body environments, the nonshedding surfaces of teeth, restorations and prostheses allow oral biofilms to maintain their attachments if left undisturbed[3].

Investigations of the links between oral and systemic diseases have focused largely on oral biofilm associated with chronic periodontitis, and on damage to the highly vascular periodontal tissues. There have been few investigations on the possible association of systemic diseases with other oral diseases and related biofilms such as carious tissue, mucosal and nonvital root canal biofilms. In particular, in persons at high risk for dental caries, investigation of possible links between carious tissue biofilms and systemic diseases should be pursued.

The purposes of the present article are to review the association between oral biofilms and systemic disease, to review relevant information regarding carious tissue biofilms and possible systemic disease, and to emphasize the critical importance of effective preventive oral care for reducing the potential for oral and systemic diseases.

## THE RELATIONSHIP BETWEEN ORAL AND GENERAL HEALTH

### Medically compromised at risk persons

Because of their deteriorating health, increasing numbers of elderly persons will reside in long-term care institutions. The presence of physical and mental disabilities, compounded by communal living, leads frequently to the neglect of oral hygiene and to major changes in diets with an increased ingestion of refined sugars. These changes, in addition to impaired saliva flow and buffering capacity arising from hyposalivation caused by many medications and dehydration, greatly increase the risk of plaque-related dental caries, periodontal disease, candidosis and associated morbidity in old age[4]. Moreover, the negative impacts of poor oral health extend well beyond the mouth[5],[6]. For example, pathogenic microorganisms that are frequently found in dental biofilms have been associated with coronary heart disease, and with chronic obstructive pulmonary disease and aspiration pneumonia in particular[7]-[14]. The latter was reported to be the leading cause of death from nosocomial infections in long-term care facilities[15].

Apart from the institutionalized, other medically at risk groups of persons include those with impaired host defense systems and pre-existing respiratory conditions who are at risk for hospital-acquired and ventilator-associated pneumonia[16]. As in nursing homes, elderly persons in hospitals also have an increased risk for pneumonia, which is not unusual and again is the leading cause of death from nosocomial infections[17].

### Bacterial biofilms

Bacterial biofilms consist of dynamic, complex three-dimensional aggregations of heterogeneous microorganisms and their sticky extracellular polysaccharide matrix, which adheres to moist mucosal and tooth surfaces and to foreign surfaces such as indwelling catheters and tubes, and dental prostheses. Biofilms are associated with, for example, brochiectasis, chronic rhinosinusitis, tonsillitis and otitis media, recurrent urinary tract infections, chronic periodontitis and dental caries[18]. Thick, persistent biofilms protect embedded slow-growing microorganisms from physical dislodgement and the effective actions of antibodies, phagocytes, and antimicrobials and antibiotics, which may then promote selective bacterial resistance[18],[19]. In some instances, biofilm aggregates may reside in a mucus layer or within the cytoplasm of epithelial cells that also could protect the microorganisms.

### Oral biofilms and systemic diseases

A recent review of possible systemic diseases linked to chronic periodontal disease concluded that although there were definite epidemiological associations, particularly for cardiovascular disease and poor glycemic control in diabetics, the associations for other medical conditions such as preterm births were not always strong[12]. Another recent systematic review and meta-analysis confirmed previous meta-analytical studies that periodontal disease is an independent, though relatively weak, risk factor or marker for coronary heart disease[14].

A microorganism found in dental plaque, *Streptococcus sanguinis*, has been reported to correlate with coronary heart disease in a Chinese population[20], and another microorganism, *Strep. mutans*, has been detected in the heart valves and blood of persons with infective endocarditis[21]. A recent study that evaluated the DNA serotype distribution of *Strep. mutans*, detected the microorganism in approximately 64% of defective heart valves and atheromatous plaques, and in 95% of dental plaques obtained from patients undergoing cardiovascular surgery[13]. High agreement was found in particular for the single serotype *e* of *Strep. mutans* from dental plaques and from defective heart valves, which contrasted with the usual serotype *c* found in the dental plaques of healthy individuals[13].

Dental plaque also has long been implicated as an important biofilm reservoir for respiratory pathogens such as *Staphylococcus aureus*, enteric Gram-negative bacilli and *Pseudomonas aeruginosa*, especially in persons with poor oral and denture hygiene and who are at high risk for aspiration pneumonia[22],[23]. Several studies have confirmed similar chromosomal DNA patterns present between microorganisms found in dental plaque, and subsequently in the lungs of many patients who developed ventilator-associated pneumonia[17],[24]-[26].

The implication from these studies is that the control of pathogens in oral biofilms may prevent such microorganisms from reaching cardiovascular and pulmonary tissues, and from potentially causing or exacerbating existing diseases.

### Oral biofilms from low and high caries risk persons

The treatment needs for high caries risk adults are approximately four times that of low caries risk adults, but current methods of prevention appear unable to effect a significant caries reduction[27]. Additionally, caries risk in the elderly is approximately twice that in children[28]. Of concern is the presence of possibly virulent pathogenic microorganisms from oral biofilms in persons having poor general health. A significantly increased risk for pneumonia has been observed in elderly persons with many carious teeth[29],[30]. Another study also reported that adults with systemic lupus erythematosus and having large carious lesions were at a significantly higher risk (odds ratio 7.5) for pneumonia compared with a similar group of adults having much better oral health[31]. From a multivariate logistic regression analysis, the numbers of carious teeth, as well as increased periodontal disease and serum lipid levels, were found to be significantly associated with acute myocardial infarction[32].

Since high caries risk persons may be linked potentially with several systemic diseases, it is important to differentiate oral biofilms formed by low caries risk persons from oral biofilms formed by high caries risk persons that may contain possible virulent pathogens. Hence, the composition of oral biofilms in the initiation and progression of caries in low and high caries risk persons requires re-evaluation[33].

Though oral biofilms are found on intact tooth surfaces, they are also implicated in the etiology of one of the most prevalent diseases, namely, dental caries[34]. The relationship between the microbiota in the biofilm and the demineralization of tooth surface suggests that certain species or combinations of species are more cariogenic than others, and that the dominance of single acidogenic species in particular is conducive to high caries activity[35].

### Oral biofilms from *in vitro* microbial models

**Fig. 1:**
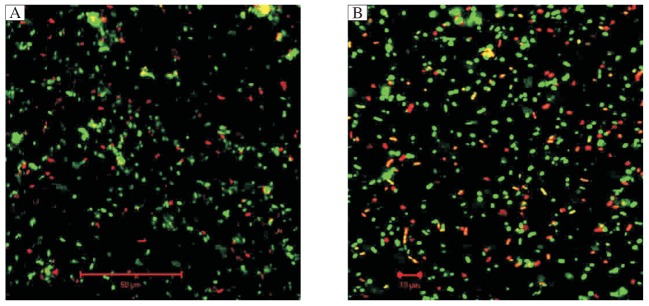
Mature oral biofilms photographed at the same magnification on d5. A: From a low caries risk person (scale bar = 50 µm). B: From a high caries risk person, showing increased live bacteria concentrations compared with those in A. Live bacteria are stained fluorescent green by SYTOR 9 stain, and dead bacteria are stained fluorescent red by propidium iodide. Note the high density of the live and dead bacteria. (scale bar = 10 µm).

It is necessary to investigate oral biofilms more closely in an attempt to identify at different ages the pathogenic microorganisms that contribute to significant differences in caries risk (***Fig. 1***). Though *in vivo* studies of artificial caries have the advantage of the actual oral environment, *in vitro* studies of artificial caries can be advantageous because most of the oral environmental conditions and the microbiota can be controlled and changed[36]. When using an artificial mouth, the development of a four species consortia biofilm of oral bacteria in a bovine enamel and dentin model system successfully reproduced dentin caries[37]. A further study using *in vitro* microbial models examined oral biofilm formed by a few known cariogenic oral microorganisms, and how they affected carbohydrate consumption and the demineralization of salivary protein-coated calcified tooth tissue[38]. Bacteria from human tooth root surface caries have also been used in an artificial mouth to investigate the effect of oral biofilms on calcified tooth tissue and restorative materials[39].

**Fig. 2:**
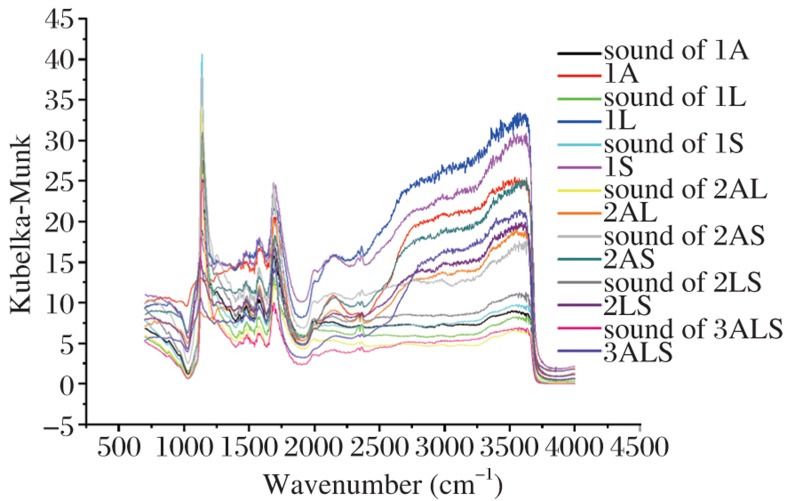
FTIR spectra of sound and demineralized root surface dentin with 1-, 2-, and 3-bacterial culture species. A: *Actino. israelii* [NT6-2A], L: *Lact. acidophilus* [ND7-2A], and S: *Strep. mutans* [ND16-6A].

Bacteria and yeasts from the mouths of high caries risk elderly persons have been studied[40]. The coaggregation of these microflora[41], and their cariogenicity in terms of root surface demineralization[42], also have been characterized. Fourier transform infrared (FTIR) spectrometry was used to calculate the intensity ratio of amide I or II to HPO_4_^2−^, to measure the degree of dentin demineralization and thus show the effects of the monoculture and co-culture of oral biofilms on human tooth root surfaces[43]. In this previous study, the cariogenicity of *Actinomyces israelii* [NT6-2A], as measured by mineral changes when compared with sound root surfaces, was the highest of all three biofilms tested (***Fig. 2***). However, because this microorganism does not appear to be associated specifically with orally related systemic diseases, further *in vitro* studies of the microflora of oral biofilms are required. Significantly, recent analysis of 1,285 bacterial 16S rDNA gene sequences from 42 persons found that almost half of the bacteria from carious lesions remain to be identified[44], and that many other novel species, apart from traditional microorganisms, may be implicated in initiating caries at different tooth sites at different ages[45].

There are significant limitations when using traditional culture, direct microscopic and biochemical methods to identify oral bacteria. Not all bacteria are cultivable or have immunological characteristics that fit into the patterns of any known genus and species[46],[47]. The use of the 16S ribosomal RNA gene in quantitative gene cloning and sequencing techniques has resulted in the reclassification and renaming of numerous bacterial genera and species and the identification of many novel bacteria. The oral cavity and the gastrointestinal tract have been the most significant sites for the discovery of potentially pathogenic novel species, including *Strep. sinensis* and many *Prevotella species* in saliva and dental plaque[46]. With at least 800 known bacterial species inhabiting the oral cavity, considerably more metagenomic research, including the use of pyrosequencing, is required to determine species significance in polymicrobial consortia infections[44],[45],[47].

### The importance of effective preventive oral care

Institutionalized persons encounter substantial barriers to dental care compared to their independent peers[48]. These include cognitive and functional disabilities, transportation problems, financial costs and anxiety[48]-[50], compounded by a lack of professional interest from physicians, dentists and dental hygienists, and a lack of facilities to treat such institutionalized persons[51]. Increased tooth retention leads to increased dental calculus and periodontal disease, and increased numbers of coronal and root surface carious lesions[52]. Consequently, studies of elderly long-term care residents in nursing homes have continued to report an increasing need for urgent preventive dental services[53]-[56].

Immunosuppressive agents and head and neck tumor irradiation may result in severe damage to the oral mucosa and salivary glands, resulting in mucosal lesions that offer a portal for systemic infection[1]. Such treatments will compromise the normal protective mechanisms of the oral cavity, creating an ecological imbalance that allows the proliferation of opportunistic microorganisms, with possible life-threatening results. A thorough oral examination of all patients, and the elimination/reduction of all sources of infection and inflammation, should be done before any medical procedures that may result in, or be compromised by, oral bacteremias.

In several studies, the use of antimicrobials such as chlorhexidine, and the application of high concentration fluorides in high caries risk elderly persons either failed to show their effectiveness in the prevention of caries[57], or met with limited success[58]-[60]. Fortunately, the oral cavity is one of the few regions of the body where thick biofilms can be readily accessed and physically disrupted. Hence, a recent review found that the focused use of tooth brushing combined with chlorhexidine rinses and gels effectively reduced the prevalence of respiratory pathogens in dental biofilms, and the rate of pneumonia in persons at high risk by approximately 40%-50%[16]. Alternatively, essentialoils containing rinses also have been shown to be very effective for the long-term reduction of oral biofilms and, unlike chlorhexidine, without staining teeth and esthetic restorations, altering taste or forming supragingival calculi[61],[62]. Additional preventive methods include the gentle use of electric tooth brushes and tongue scrapers, and the use of sugarless chewing gums and casein-derived pastes/cremes. It is essential also that adequate hydration and saliva flow be maintained. Good denture hygiene is also very important for reducing potential pathogens present in oral biofilms[63].

## CONCLUSION

There is increasing evidence of the important relationship between oral biofilms and systemic diseases such as cardiovascular, central nervous system, respiratory and skeletal infections, and to nosocomial infections that are associated with a high morbidity in institutionalized and hospitalized elderly persons in particular. Microorganisms from oral biofilms have also been identified by DNA genotyping in defective heart valves and atheromatous plaques. Persons who have many, acute carious lesions caused by virulent microorganisms may be at a greater potential risk for several systemic diseases, particularly if their general health is poor. This possible high risk association requires further metagenomic and clinical research. The physical disruption of thick oral biofilms is necessary to allow the effective action of antimicrobials.
